# Capacity building, local ownership and implementation of a multi-level HIV/AIDS positive health, dignity, and prevention initiative in Mozambique: approach, challenges and lessons learned

**DOI:** 10.1080/16549716.2020.1769900

**Published:** 2020-07-03

**Authors:** Carol Dawson-Rose, Sarah A. Gutin, Elsa Hunguana, Florindo Mudender, Sebastian Kevany

**Affiliations:** a Department of Community Health Systems, School of Nursing, University of California, San Francisco, CA, USA; b I-TECH Mozambique, University of Washington, Maputo, Mozambique

**Keywords:** HIV/AIDS, prevention with positives, local ownership, Mozambique, positive health, dignity and prevention interventions

## Abstract

Mozambique has for many years suffered from a high burden of HIV with an estimated prevalence of 11.1% among adults age 15–49 years. In response, Positive Health, Dignity, and Prevention (or Positive Prevention as it is known in Mozambique), was developed as a method of integrating HIV care and prevention via capacity building. Through comprehensive holistic care, HIV transmission is prevented while simultaneously promoting the health of people living with HIV/AIDS. Our initiative used a three-tiered approach, and included activities at national, provincial, and community levels. In order to change patient behavior and successfully train health-care workers in Positive Prevention, it was therefore considered necessary to work at multiple levels of influence. This ensured that the individual-level behavior change of PLHIV and health-care providers was maximized through supportive environments and policies. Related national-level achievements included the establishment of a Positive Prevention technical working group; the development of a Positive Prevention policy document; training national policy-makers on Positive Prevention; the development and distribution of a nationally approved Positive Prevention training package; the integration of Positive Prevention into existing Ministry of Health curricula; the development and approval of national data collection forms; and the drafting of a related national strategy. The framework and key activities of the Mozambique Positive Prevention Program may help to inform and assist others involved in similar work, as well as advancing country or local ownership of HIV/AIDS treatment, care and prevention efforts. By using a three-tiered approach, a supportive system was created. This was critical to both optimizing Positive Prevention provision and building long-term capacity. In order for related efforts to be successful in other settings, we encourage implementing partners to also work at multiple levels, with local ownership principles in mind, in order that Positive Prevention programs may have the greatest possible effect.

## Background

In 2012, Mozambique suffered from the 8th highest HIV prevalence in the world with an estimated prevalence of 11.1% among adults aged between 15 and 49 years [1]. At that time, some provinces in Mozambique reported HIV prevalence rates as high as 25% [2]. In response, Positive Health, Dignity, and Prevention (or Positive Prevention [PP], as it is known in Mozambique), was developed as a method of integrating HIV care and prevention. The aim of PP was to prevent HIV transmission while simultaneously promoting the health of persons living with HIV (PLHIV) by offering comprehensive holistic care.

PP has been included as a core component of HIV care in many countries receiving support from the US President’s Emergency Plan for AIDS Relief (PEPFAR). The PP package of services includes: monitoring antiretroviral treatment (ART) adherence or treatment as prevention; assessment and treatment of sexually transmitted infections (STIs); assessment of partner status and HIV disclosure to partners; family planning and reproductive health counseling; provision of condoms and lubricants; and referral to community-based programs for non-clinical support (e.g. behavioral prevention support, food and nutrition programs) [3].

To this end, the International Training and Education Center for Health (I-TECH), a collaboration between the University of California, San Francisco (UCSF) and the University of Washington (UW), began implementing a PP initiative in Mozambique in 2011. Through partnership with the government of Mozambique (GoM), the U.S. Centers for Disease Control and Prevention (CDC) Global AIDS Program, and other local organizations, an innovative and tailored PP program was adopted for implementation. The Mozambique PP program focused not only on the above-mentioned package of services, but also on long-term capacity building and health system strengthening. This PP program was integrated into HIV treatment efforts across Mozambique [4], with the aim of transitioning from a technical assistance model to full country ownership and sustainability of the program [5]. Our aim is to describe the framework, procedures, adaptations, and key activities of the Mozambique PP program implementation. We also describe the capacity building partnership and the transition to Mozambican ownership and management of service delivery.

### Theoretical basis for locally owned PP approaches

**Figure 1.**
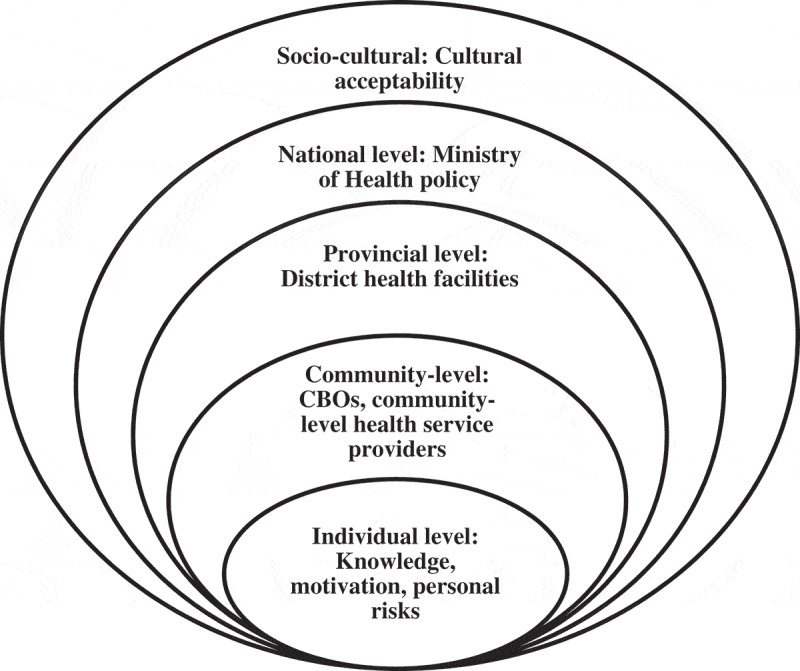
The socioecological model for capacity building in geographical context.

Capacity building is built on partnerships and collaborations, as well as on how such efforts can be sustained in low- and middle-income countries (LMICs). The PP program initiative used a three-tiered framework based on the socioecological framework that included activities at national, provincial, and community levels (see Figure 1) [6]. Briefly, the approach emphasizes the interaction between, and interdependence of, factors that affect a health problem across multiple levels [7]. In order to address individual care and behavior, successfully train health-care workers in PP, and guide provincial and national level procedures it was necessary to work at multiple levels (i.e. national, provincial, and community). This helped to ensure that the individual-level behavior change of PLHIV and health-care providers was maximized through supportive environments (e.g. buy-in at all levels of the health system) and policies.

The socioecological model as presented thus recognizes that individuals are nested within systems and environments. PP implementation aimed to improve the individual knowledge and skills of providers while simultaneously building supportive systems at the organizational (e.g. district and provincial health structures), community, and at national levels. In Mozambique, the PP program was adapted for country level acceptability, as described elsewhere [8]. Stakeholders including national Mozambican government and provincial level stakeholders collaborated to train health-care service providers and other community level partners in advancing capacity building efforts as described in the approach detailed below.

## Methods

### PP strategy, approach and components

As originally conceived in 2010, the vision of the Mozambique PP Program was to ensure that, within five years, the Ministry of Health (MOH) would have the capacity and systems at national, provincial and district levels to: firstly, support the integration of PP into patient visits and referral systems within health facilities, health-care provider training, health facility data collection, and quality improvement tools; secondly, support HIV community groups to create and sustain linkages between health services and a range of community-based services; and, thirdly, develop a cadre of clinical and community-based trainers and focal persons to implement effective PP training and ensure that PLHIV were linked with both facility and community-based services.

Efforts to achieve these goals took place through a three-pronged approach to PP that included an emphasis on training, integration, and system strengthening. For maximum impact in Mozambique, this approach was implemented concurrently at the national (central government), provincial, district (health facility), and community levels. This helped to ensure that, wherever patients entered the health system, PP information and services would be offered.

Finally, central to the PP approach was the belief that health facilities should be the focal point of PP efforts and implementation. For example, at the district level, health facilities often serve as PP entry points via service providers and clinic focal points liaising with community-based organizations (CBOs) and other community-level health service providers. Health facilities also serve as ideal venues to host coordination meetings between health facility staff and community leaders to discuss referrals of PLHIV both to and from (and also within) facilities, as well as their public sector tasks of HIV counseling and testing services, maternal and child health, and HIV treatment.

The PP standard of care in Mozambique included eight key components similar to the essential services recommended by PEPFAR: sexual behavior risk assessment and provision of condoms; partner testing and disclosure counseling; ART adherence counseling; STI testing and treatment; family planning and prevention of mother-to-child transmission (PMTCT); alcohol use assessment and counseling; referral to community-based support services; and assessment and counseling for gender-based violence (GBV).

## Capacity building results

### National-level system change

Under multiple-level approaches such as those employed in this case, the strength of the health facility and community providers is considered most effective in an enabling, supportive policy environment, led by leadership from the national level. Related national-level activities, therefore, included the establishment of a PP technical working group (TWG); the development of a PP policy document; training national policy-makers on PP; the development and distribution of a nationally approved PP training package; the integration of PP into existing MOH curricula; the development and approval of national data collection forms; and the drafting of a related national strategy, all of which are demonstrable elements of program contributions to local long-term capacity building.

### The PP technical working group

With guidance and support from UCSF, I-TECH, and the CDC, the MOH PP TWG was established in April 2011 with a mandate of integrating PP into key policies, procedures and existing MOH curricula for clinical and non-clinical public health service staff. The group met regularly, and three subgroups were formed to focus on: PP integration within health units; PP at the community level; and monitoring, evaluation, and documentation. Shortly after its formation, TWG members also endorsed a standard of care for PP that was subsequently accepted by the MOH. Such ministerial buy-in was an important step towards further legitimizing PP efforts in Mozambique, and reinforced the importance of both integrating PP messages in health services; the need to implement PP at health facility and community levels to ensure consistency in prevention information; and the need to combine such efforts with broader consideration of capacity building efforts and consequences in a scientific and demonstrable manner. In addition, the structure of the TWGs was in keeping, from a capacity-building perspective, with past efforts to develop sustainable organizational capacity in resource-constrained settings [9].

After the formation of the PP TWG, it was suggested that a psychosocial support element be integrated with PP. As a result, the national TWGs for PP and Psychosocial Support (known locally as APSS) merged into one group. Subsequently, APSS and PP built a common strategy and developed integrated training materials and monitoring and evaluation (M&E) instruments. The joint group also benefited from leadership from MOH, as well as active participation from government, donor and other non-governmental organization stakeholders.

### PP policy development; training national policymakers on PP

A PP feasibility and acceptability study among PLHIV and health-care providers was also conducted. Based on an analysis of data from the study, a policy document for the MOH was compiled [10]. A final policy document received both MOH and CDC approval, and was then distributed to health officials nationwide. As part of the PP program, national-level MOH officials were also trained on PP in order to encourage its inclusion at both the policy and health facility levels. Three complimentary activities took place: UCSF/I-TECH was requested by the National Department of Medical Assistance (DNAM) to present the results of the PP feasibility and acceptability study [11]; a policy document was circulated among MOH stakeholders; and, thirdly, a one-day meeting on PP was attended by officials from the MOH and other stakeholder organizations. Cumulatively, these activities helped to orient MOH and non-governmental stakeholders on PP; created a platform for sustainability and local ownership of program design and delivery; and contributed to capacity building in similar ways to mentored fellowship systems in other sub-Saharan African countries [12].

### PP training packages

Through the TWG, UCSF/I-TECH worked closely with local partners (e.g. Prevenção Activa e Comunicação Para Todos [PACTO], or Active Prevention and Communication for All) to develop PP training materials for activities at health facility and community levels, via associated informal formative research at field level. This close collaboration also allowed for the harmonization of messages between facility-based PP and community support services. Facility-based training materials were composed of a facilitator’s guide; a participant’s manual; a reference manual; a flip chart; a poster; and a pocket guide for providers. Community materials were also developed for community health workers and activists and comprised a facilitator’s manual, a participant’s manual, and a flipchart. This training package was subsequently approved by the MOH, and represented a significant step towards standardizing PP content and materials.

### Integration with existing materials and curricula

Another national-level task was integrating PP within existing trainings and curricula. This included integration of PP into national pre-service training for medical technicians; in-service training curricula; clinical mentoring programs for health-care providers that later became part of the national Quality Improvement and Humanization Initiative (QIHI) through the MOH; and a maternal and child health (MCH) nurses task-shifting training (developed to increase nurse capacity to manage HIV treatment as part of the expansion of lifelong ART for all pregnant women through the Option B+ strategy). As noted above, APSS and PP training packages were also integrated to create a single cohesive APSS/PP training approach. Of note, all of these materials had a strong equity orientation (gender, region), in keeping with many information led capacity building efforts [13].

### Data collection tools

Prior to these initiatives, no nationally approved data collection forms on which to capture PP indicators existed. Through the TWG, related forms for APSS/PP were developed and piloted at 10 health centers in three provinces (Gaza, Zambézia and Cabo Delgado). Providers at each of the pilot centers received theoretical and practical training on how to record APSS/PP interventions, and also received TA visits on how to fill out related M&E forms (Mozambique Ministry of Health, personal communication, 2015). These supervision visits were also used to gather data, standardize data collection, and identify provider gaps in use of forms. Of note, the final official MOH APSS/PP registers are currently being used in all facilities offering APSS/PP services.

## Discussion

### PP implementation and integration at provincial and district levels

An integrated and sustained approach was central to APSS/PP, and related initiatives, under both the local (or country) ownership paradigm and the above definition of capacity building. The presence of provincial PP coordinators was key to developing such an approach, and reflected the national-level system change efforts described above. Amongst other duties, provincial coordinators provided technical support for the integration of PP within existing points of care at heath facilities (e.g. antenatal, PMTCT, family planning, post-natal, and tuberculosis care); and provided system strengthening by supporting the development of provincial TWGs. PP Coordinators also transferred knowledge and skills on PP to health officers at the provincial and district levels through the provision of trainings and on-going technical support in pursuit of a ‘full site approach”, whereby patients received PP messages at all points of care. PP coordinators were also responsible for facilitating monitoring visits with local MOH representatives; integrating PP staff into the Provincial Health Directorate; supporting the Provincial Health Directorate in the coordination of PP activities; establishing provincial TWGs; providing trainings at the provincial level using nationally approved materials and job aids; and providing technical assistance to health centers.

### PP staff integration at provincial level

The development of PP training packages described in the results section above included coordinators being hired and supported by UCSF/I-TECH in the above three target provinces, while the Maputo-based PP team supported facility-based PP implementation in Maputo City and province. In geographical areas where prevalence was higher than the national average, a focus on strong relationships with Provincial Health Directorates were also established. Provincial coordinators were phased in over time, and all provincial coordinators were hired in consultation with Provincial Health Directorates. Of note, the provincial PP coordinators were housed in Provincial Health Directorates to assure MOH involvement in the scale-up of PP activities. As members of Provincial Health Directorate staff, provincial coordinators also played a role in provincial annual program planning by joining provincial management committee meetings. At these meetings, attendees discussed goals and targets for the year, difficulties encountered and possible solutions, upcoming activities, and expansion of ART to other health centers.

### TWGs at provincial level

A key capacity-building result of our work was the PP TWG (as described above). PP coordinators were then responsible for establishing and facilitating provincial-level PP TWGs to include representatives from provincial and district directorates of health, health facilities, NGO partners, and HIV group representatives. Initiating PP TWGs in the provinces required both support from Provincial Health Directorates and local buy-in: in each province, approval for the formation of a TWG was first required from the local medical chief. In each target province, TWG meetings were attended by representatives from Provincial Health Directorates, the Provincial APSS/PP focal person, the provincial HIV/AIDS supervisor, NGO partners, and local HIV organizations. TWG meetings centered around the PP intervention strategy; implementation; management and coordination of APSS/PP activities; harmonization of key APSS/PP messages to be communicated to patients; and coordination of referrals and counter-referrals between facility and community. Over time, the provincial TWGs served as a forum for discussing APSS/PP activities within the provinces, for planning trainings on the use of the new APSS/PP instruments; and for mapping the training needs of frontline nurses and other facility level health workers.

### Training at provincial level

PP policy development and training national policymakers on PP (as described in the results section above) also led to PP provincial coordinators becoming responsible for planning roll-out of training and support activities in their target province, and for identifying any constraints or concerns that might affect program implementation. In the target provinces, over 3,892 health-care workers were trained in PP over a five-year period. PP training focused on in-service/on-the-job trainings at ART and pre-ART centers in order to avoid delays in patient care. Seventy on-the-job in-service PP training events were conducted for nurses, MCH nurses, midwives, medical technicians, other medical agents, physicians, psychologists, psychiatric technicians, counselors, peer educators, and laboratory and pharmacy technicians. Training methodology also focused on using clinical cases as a way to both understand PP and incorporate the strategy into regular care for patients. Feedback suggested that trainees felt the trainings allowed for active participation in sessions, the expression of personal experiences, and asking questions; and integration with existing materials and curricula (described above).

### Technical assistance at facility level

After the training-based focus of the first two years of the program, operational research focused on whether trained providers gained PP skills over time and were independently capable of integrating and providing PP standards of care. Therefore, the aim of TA visits evolved to strengthening the capacity of providers to implement PP during regular interactions with PLHIV. In order to systematize these visits across provinces, TA orientation guides and associated data collection tools were created. The data collection tools described in the result section above were also key to this process. Related TA visits began in early 2013, for 153 health-care workers (106 clinicians and 47 non-clinicians) in 39 health centers (8 in Zambézia, 7 in Gaza, 6 in Inhambane, 8 in Maputo City, and 10 in Maputo Province). In total, providers received 5 rounds of on-site TA during which 662 visits were completed, and 1,955 consultations observed. Between rounds of follow-up, 27% of providers were lost to follow up. A second round of longitudinal TA was later launched at 6 additional health centers, at which 528 consultations were observed with 56 health-care providers.

### Integration at community level

PACTO worked closely with UCSF/I-TECH at the facility level in order to ensure appropriate adaptation and implementation of PP at community-level. UCSF/I-TECH also worked closely with PACTO and other partners to ensure adaptation and implementation of PP into community-level PP activities; both UCSF/I-TECH and PACTO shared facility and community-based PP materials and offered mutually supportive TA when needed. In provinces where community implementation partners such as PACTO were not active, PP provincial coordinators met with representatives from NGOs, CBOs, and HIV associations to develop linkages and patient referral systems. Of note, a number of implementing partners suggested both that peer educators were also required and that community members should be trained on PP.

## Challenges and lessons learned

### Community level

At an early stage of the development of a national PP roll-out strategy, PEPFAR representatives in the PP TWG prioritized health facilities as the focal point of PP implementation. Their focus on health facilities meant that community implementation efforts to work with PLHIV often lacked a standardized approach, as well as related monitoring and evaluation systems (as described above). In this regard, the decision to focus on health facilities was a key lesson learned based on the success of similar HIV prevention interventions in high-income settings. These PP clinic-based initiatives have demonstrated a reduction in the amount of risk behavior reported by HIV-positive persons in care [14], and were found to be both acceptable and feasible for clinic implementation [15].

The TWG also faced challenges regarding competing ideas and priorities about the best way to integrate PP in communities and clinics and create linkages between the community and health facility for PLHIV. Although all stakeholders agreed on the importance of PP and the need to integrate PP between health facilities and communities, the lack of a unified approach from all partners created challenges across related continuums of care and represented a further key lesson learned at the community level.

### Facility level

At the health facility level, the challenge of integrating PP was driven by perceptions of PP as falling into the realm of psychosocial support. This meant that clinicians often did not discuss PP because they saw it as outside their clinical purview; in response, by combining PP and clinical mentoring activities at UCSF/I-TECH, PP competencies were integrated into other MOH-supported clinical trainings. Clinical staff, including MCH staff, were thus provided with PP messages as part of in-service trainings, representing a key facility-level lesson learned. During these events, evidence about HIV treatment as prevention and the importance of clinicians providing PP interventions was a key focus. Finally, the initiation of PP TWGs at the provincial level was often challenging (as described above), requiring both Provincial Health Directorate and local approval, although such procedures were viewed as worthwhile in their advancement of the country or local ownership model in Mozambique.

### Cultural and historical context

One of the challenges in implementation of global health initiatives in many countries is the lack of representation of local level leaders and citizens in policy development [16]. Despite this, related research has suggested a strong sense of group-based citizenship in Mozambican communities, rooted in the country’s mass mobilization movements of the 1970 s and 1980 s (and reinstituted through post-war decentralization policies) [17], allowing for community representation and participation. Conversely, this has also been presented as a means of top-down governance that stresses what traditional leaders can do for the state, rather than their roles as representatives of their communities [17]. Taking this context into consideration, working with stakeholders and leaders at all levels was both a key lesson learned and critical to the forward movement and adoption of the PP program as a national strategy; integration in clinical forms; and in community and individual provider level skills training initiatives being adopted by other CBOs and NGOs.

## Conclusion

Much work on PP capacity-building efforts in Mozambique continues to await approval at the local or in-country level. This includes the creation of an accredited cadre, similar to peer educators or case managers, in order to integrate all components and develop a standardized training package. Yet, at the time of press, UCSF/I-TECH has been charged with the development of a related cadre based on a health educators’ strategy. This includes an integrated and locally developed training package currently undergoing its final stage of revision by the MOH related to HCT; PP; GBV initiatives, mother support groups; enhanced partner services (that include disclosure to partners); and tuberculosis treatment and care. This is planned, at health facility and community levels, to focus on care and treatment, retention, disclosure of HIV status, and notification and testing of sexual partners and family members by HIV-positive persons. Key audiences include clinicians (e.g. nurses, clinical officers, physicians) and psychosocial agents such as counsellors, peer educators, activists, and psychiatrists.

While a more unified PP approach where a single partner oversees the community and facility implementation may have been advantageous from the capacity-building perspective, the framework as described here and the key activities of the Mozambique PP Program may help to inform and assist others involved in similar capacity building initiatives. By using a three-tiered approach, a supportive system at multiple levels of influence in Mozambique was created; this was critical to maximizing the provision of PP by health-care providers. In order for related efforts to be successful in other settings, we encourage implementing partners to also work at multiple levels, with local ownership principles in mind, in order that PP programs may have the greatest possible effect.

## Paper context

Little was known about the transition from international to local ownership of HIV/AIDS programs in the Mozambique/positive prevention context prior to our research. We found that local (or country) ownership can successfully take place as long as the appropriate transition and sustainability elements are legislated for throughout the intervention. The implications of this are important for the design and delivery of other related programs, in Mozambique and elsewhere.
